# Editorial: Application of Systems Biology in Molecular Characterization and Diagnosis of Cancer

**DOI:** 10.3389/fmolb.2021.668146

**Published:** 2021-05-28

**Authors:** Cheng Zhang, Yongjun Wei, Adil Mardinoglu, Peng Zhang

**Affiliations:** ^1^Science for Life Laboratory, KTH - Royal Institute of Technology, Stockholm, Sweden; ^2^School of Pharmaceutical Sciences & Key Laboratory of Advanced Drug Preparation Technologies, Ministry of Education, Zhengzhou University, Zhengzhou, China; ^3^Faculty of Dentistry, Oral & Craniofacial Sciences, Centre for Host-Microbiome Interactions, King's College London, London, United Kingdom; ^4^Department of Surgery, School of Medicine, University of Maryland, College Park, MD, United States

**Keywords:** systems biology, cancer, multi omics, cancer diagnoses, molecular subtype classification

Cancer is one of the top killers of human beings, causing ~10 million deaths in 2020 alone, which makes it either the first or second leading cause of death for people under 70-years-old (Sung et al., 2021). Therefore, there is an increasing need for an effective diagnosis and treatment for cancer, and researchers have spent a huge amount of resources and efforts to define the molecular mechanisms driving its development and progression. However, studying cancer is quite difficult as there is huge heterogeneity among human cancers, which means the variation of different individuals diagnosed with the same cancer type can sometimes be greater than that of patients from different types of cancers (Uhlén et al., 2017). As a result, most current cancer drugs are only effective in a certain subgroup of patients, and there is still a huge gap in our understanding of other treatment approaches and cancer pathogenesis (Brennan et al., 2010).

To address this huge heterogeneity among cancer patients, there is an urgent need to develop personalized diagnostic strategies to characterize cancer patients with different molecular profiles which could consequently facilitate the development of personalized and precision medicine for better treatment strategies of individual cancer. Systems biology has been a powerful tool in the integration of omics data and the characterization of different cancers. The cancer research community is increasingly using systems biology approaches to understand the complex molecular profile of cancers and decipher the mechanisms of tumor progression for the development of more effective cancer therapies (Du and Elemento, 2015). Creighton et al. characterized four different subtypes of clear cell renal cell carcinoma based on multi-omic molecular profile of the tumor (Creighton et al., 2013). Bidkhori et al. employed a metabolic network to stratify hepatocellular carcinoma and revealed three molecular subtypes relying on alternative enzymes to catalyze the same metabolic reactions (Bidkhori et al., 2018). In addition, Toy et al. performed a meta-analysis on long non-coding RNA HOX transcript antisense RNA using publically available data and identified potential prognostic biomarkers for the prediction of the survival of different cancers (Toy et al., 2019). In this Research Topic,  Shi et al. reviewed the recent progression of multi-omic data integration for the study of gastric cancer. The authors specifically focused on systems biology approaches for integration of multi-omics data, and also discussed the association between gastrointestinal microbiota and gastric cancer. In addition, Zhang et al. summarized recent understanding of four existing molecular subtypes of glioblastoma, and the indication of these classifications in guiding diagnosis, prognosis, and treatment of cancer.

Thanks to the rapid development of Next-Generation Sequencing technology, the cost and time needed for the generation of RNA-sequencing data have been significantly reduced in the past few years. As a result, a huge amount of transcriptomic data has been generated from different cancer patients and made publically available, which greatly facilitated the molecular characterization of subtypes based on cancer transcriptomic profile. In this context, many transcriptomic based models have been developed for the diagnosis of cancer molecular subtypes. Hu et al. evaluated the transcriptomic profile of tumor and adjacent normal tissue samples as well as lymph nodes from Head and Neck Squamous Cell Carcinoma patients, and identified a list of gene markers for metastasis of the tumor. Based on the transcriptomic profiles of gastric cancer patients, Dai et al. revealed the association between mucins and clinical outcomes. In addition, they proposed a prognostic marker by combining the transcriptomic expression of two mucin related genes. Kaushik et al. who are more interested in non-small cell lung cancer, focused on the commonly differentially expressed genes among several patient cohorts, and proposed a combined prognostic model which can stratify patients into different molecular subgroups with different survival outcomes.

The canonical transcriptomic and survival analyses are sensitive to the batch effect, and they may also mask the heterogeneity of individual cancer patients. In order to address these issues, Chen et al. and Guan et al. both applied a method (Wang et al., 2015) that uses gene ranking within each individual sample for patient classification to study the molecular subtypes of breast cancers. Both of these studies obtained biomarkers that could classify the individual patient into different subtypes which are either resistant or sensitive to a certain treatment.

Apart from transcriptomics, other omics profiles can also be integrated and applied in characterizing cancer molecular subtypes.  Xu et al. integrated both genetic and transcriptomic information from breast cancer patients and identified immune subtypes among the patients. Wu et al. analyzed both proteomics and transcriptomics data from patients and identified XRCC1 as a promising predictive biomarker and therapeutic target for gallbladder cancer. Deng et al. integrated clinical and comprehensive molecular information from patients diagnosed with endometrial carcinoma and built a prognosis model to predict the prognosis of the patients from different identified subgroups. Elnemr et al. developed a machine learning method to identify biological causes of malignant diseases based on protein correlations. Moreover, Cheng et al. reported that cancer purity correlates with the number of mutations in tumors and will affect the genomic mutation profile in pathological analyses.

The work presented in this Research Topic highlights the importance of the characterization of the molecular subtypes of different cancers and presents many recent studies that identify different cancer subtypes based on transcriptomics, proteomics, and/or genomics using systems biology approaches. These studies provide valuable insights and extend understanding of the complexity of cancer pathogenesis and progression, and will accelerate the development of personalized and precision medicine for cancer treatment.

## Author Contributions

CZ, YW, AM, and PZ wrote the editorial together and approved its final version. All authors contributed to the article and approved the submitted version.

## Conflict of Interest

The authors declare that the research was conducted in the absence of any commercial or financial relationships that could be construed as a potential conflict of interest.

## References

[B1] Bidkhori G. Benfeitas R. Klevstig M. Zhang C. Nielsen J. Uhlén M.. (2018). Metabolic network-based stratification of hepatocellular carcinoma reveals three distinct tumor subtypes. *Proc. Natl. Acad. Sci*. U.S.A. 115, E11874–E11883. 10.1073/pnas.1807305115 PMC6294939 30482855

[B2] Brennan D. J. O'Connor D. P. Rexhepaj E. Pontén F. Gallagher W. M. (2010). Antibody-based proteomics: fast-tracking molecular diagnostics in oncology. Nat. Rev. Cancer 10, 605–617. 10.1038/nrc2902 20720569

[B3] Creighton C. J. Morgan M. Gunaratne P. H. Wheeler D. A. Gibbs R. A. Muzny D.. (2013). Comprehensive molecular characterization of clear cell renal cell carcinoma. The Cancer Genome Atlas Research Network. Analysis Working Group: Baylor College of Medicine. Nature 499, 43–49. 10.1038/nature12222 23792563 PMC3771322

[B4] Du W. Elemento O. (2015). Cancer systems biology: embracing complexity to develop better anticancer therapeutic strategies. Oncogene 34, 3215–3225. 10.1038/onc.2014.291 25220419

[B5] Sung H. Ferlay J. Siegel R. L. Laversanne M. Soerjomataram I. Jemal A.. (2021). Global cancer statistics 2020: GLOBOCAN estimates of incidence and mortality worldwide for 36 cancers in 185 countries. CA A Cancer J. Clin. 71, 209–249. 10.3322/caac.21660 33538338

[B6] Toy H. I. Okmen D. Kontou P. I. Georgakilas A. G. Pavlopoulou A. (2019). HOTAIR as a prognostic predictor for diverse human cancers: a meta- and bioinformatics analysis. Cancers 11:778. 10.3390/cancers11060778 31195674 PMC6628152

[B7] Uhlén M. Zhang C. Lee S. Sjöstedt E. Fagerberg L. Bidkhori G.. (2017). A pathology atlas of the human cancer transcriptome. Science 357:eaan2507. 10.1126/science.aan2507 28818916

[B8] Wang H. Sun Q. Zhao W. Qi L. Gu Y. Li P.. (2015). Individual-level analysis of differential expression of genes and pathways for personalized medicine. Bioinformatics 31, 62–68. 10.1093/bioinformatics/btu522 25165092

